# Itch: from the skin to the brain – peripheral and central neural sensitization in chronic itch

**DOI:** 10.3389/fnmol.2023.1272230

**Published:** 2023-10-02

**Authors:** Omar Mahmoud, Olusola Oladipo, Rami H. Mahmoud, Gil Yosipovitch

**Affiliations:** Dr. Phillip Frost Department of Dermatology and Cutaneous Surgery, Miami Itch Center, University of Miami Miller School of Medicine, Miami, FL, United States

**Keywords:** neural sensitization, chronic, itch, peripheral, central, nervous system, brain, pruritus

## Abstract

Similar to chronic pain, chronic itch is frequently linked to neural sensitization, a phenomenon wherein the nervous system becomes hypersensitive to stimuli. This process of neural sensitization of chronic itch is orchestrated by various signaling pathways and mediators in both the peripheral and central nervous systems. At the level of the peripheral nervous system, inflammation and neuroimmune interactions induce plastic changes to peripheral nerve fibers, thereby amplifying the transmission of itch signaling. Neural sensitization in the central nervous system occurs at both the spinal cord and brain levels. At the level of the spinal cord, it involves hyperactivity of itch-activating spinal pathways, dysfunction of spinal inhibitory circuits, and attenuation of descending supraspinal inhibitory pathways. In the brain, neural sensitization manifests as structural and functional changes to itch-associated brain areas and networks. Currently, we have a diverse array of neuroimmune-modulating therapies targeting itch neural sensitization mechanisms to help with providing relief to patients with chronic itch. Itch research is a dynamic and continually evolving field, and as we grow in our understanding of chronic itch mechanisms, so will our therapeutic toolbox. Further studies exploring the peripheral and central neural sensitization mechanisms in the context of chronic itch are needed.

## 1. Introduction

Itch is an unpleasant sensory phenomenon that is encoded by histaminergic (in acute cases) and nonhistaminergic (in the majority of chronic cases) neuronal pathways (Yosipovitch et al., 2018). The pathophysiology of chronic itch, often defined as itch lasting greater than 6 weeks, involves crosstalk between keratinocytes, the immune system, and sensory neurons (Yosipovitch et al., 2018). Chronic itch is often associated with neural sensitization, which describes the process by which the nervous system experiences heightened sensitivity to stimuli. Alloknesis and hyperknesis represent manifestations of neural sensitization. Alloknesis is defined as normal or nonpruritic stimuli inducing the sensation of itch, and hyperknesis refers to excessive itch perception to a pruritic stimulus (Yosipovitch et al., 2018).

Neural sensitization is involved in chronic pain and itch conditions, both of which share common features including sleep abnormalities, fatigue, and psychological disturbances, and both involve peripheral and central neural sensitization (Yosipovitch et al., 2007). In chronic itch, ongoing activation of sensory nerve fibers by pruritogenic cytokines and other mediators promotes neurogenic inflammation, neuronal plasticity, and sensitization of nerve fibers resulting in dysregulation of neuroimmune circuits and persistent itch (Steinhoff et al., 2022; Tominaga and Takamori, 2022). In contrast to chronic pain, chronic itch conditions often lead to a vicious itch-scratch cycle, whereby scratching sustains the inflammatory response via skin barrier disruption, further promoting sensitization (Mack and Kim, 2018). Atopic dermatitis (AD) is a prototypical example of this process. In one study, pruritogen-sensitive C-fibers recorded in AD patients exhibited high levels of spontaneous firing (Andersen et al., 2017). Moreover, we found increased susceptibility to both cowhage and mechanically-evoked itch, particularly intralesionally, in AD patients, a finding suggesting involvement of sensitization of the non-histaminergic pathway as well as mechanosensitive circuitry not normally associated with itch (Andersen et al., 2017; Yosipovitch et al., 2020). AD has central sensitization in which stimuli that are perceived as painful in healthy subjects are experienced as itching in AD patients; not only specific allergens, but also stress and psychosocial factors can exacerbate AD symptoms. Other itchy conditions such as psoriasis, neuropathic itch, and chronic pruritus of unknown origin exhibit features of peripheral and central neural sensitization. Notably, we recently reported that prurigo nodularis is highly linked with the common chronic pain neural sensitization disorders fibromyalgia, interstitial cystitis, and irritable bowel syndrome, in particular, with a 24-fold higher risk for interstitial cystitis, supporting a common mechanism of neural sensitization in all of these disorders (Choragudi and Yosipovitch, 2023).

**Figure 1:**
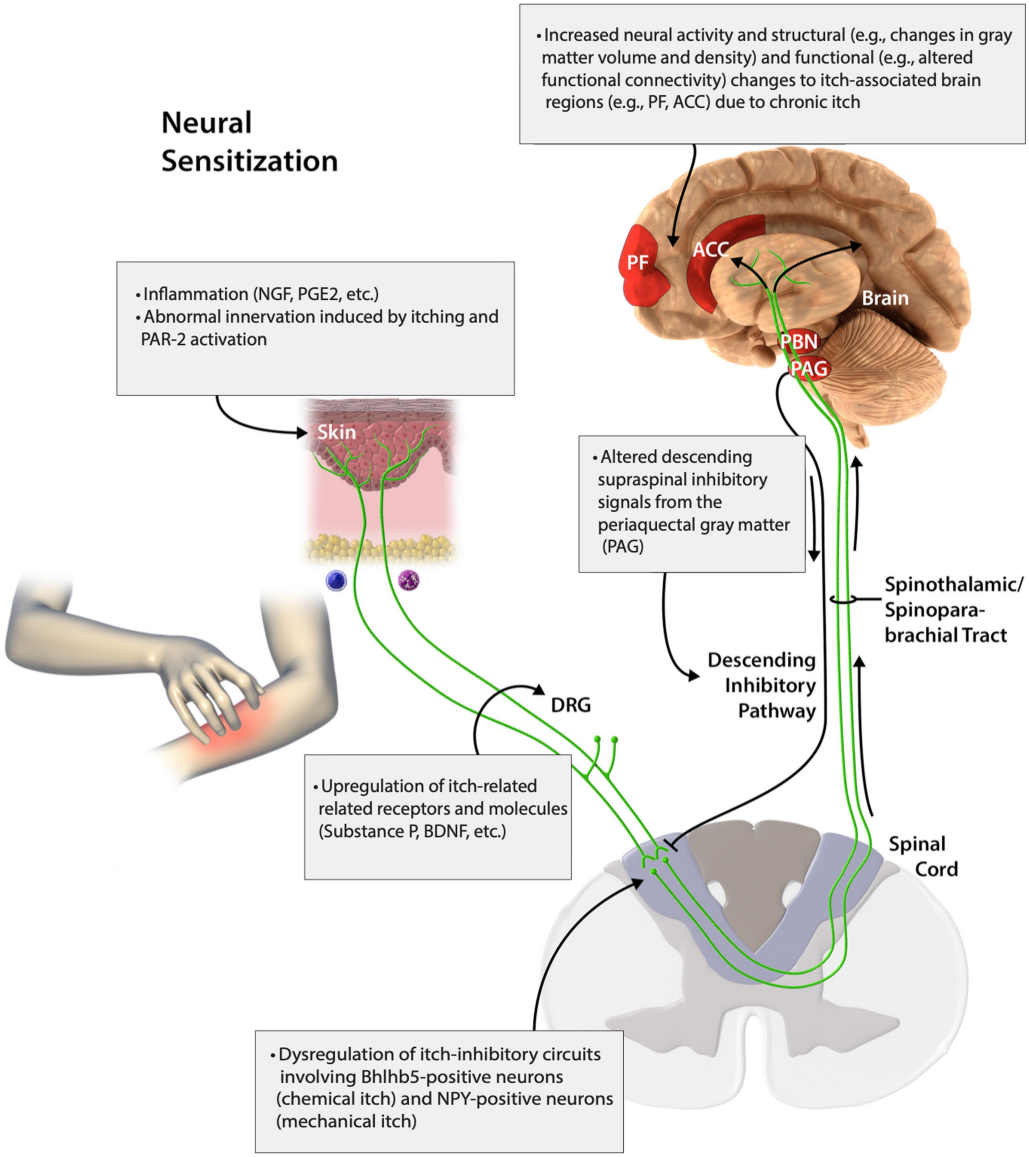
Peripheral and central neural sensitization of itch. Adapted with permission from Yosipovitch et al. (2018). This figure was published in J. Allergy Clin. Immunol, 142, Yosipovitch, G., Rosen, J. D., and Hashimoto, T., Itch: from mechanism to (novel) therapeutic approaches., 1375–1390, Copyright Elsevier (2018).

From the skin to the brain, there are several mediators of this process. At the cutaneous level, inflammation, PAR-2 activation, and disordered innervation induced by inflammation and scratching all represent key mediators of neural sensitization (Yosipovitch et al., 2018). Of particular interest is nerve growth factor, a neurotrophic factor released predominantly by epidermal keratinocytes and eosinophils responsible for elements of peripheral sensitization (Yosipovitch et al., 2018). Upregulation of substance P, a peripherally and centrally active neuropeptide released by nerve fibers and stimulated proteases that causes neurogenic inflammation, brain-derived neurotrophic factor, and other itch-inducing molecules and receptors contribute to sensitization at the dorsal root ganglion (Yosipovitch et al., 2018; Vander Does et al., 2023). At the level of the spinal cord, dysfunction of inhibitory circuits including neuropeptide Y and Bhlhb-5 neurons, as well as attenuation of descending, inhibitory pathways are at play. In the brain, there are structural and functional changes that can perpetuate the sensation of itch (Yosipovitch et al., 2018). In this review, we evaluate the key mechanisms and mediators of neuronal sensitization and how they contribute to the experience of itch sensation (Figure 1).

## 2. Peripheral neural sensitization in chronic itch

Peripheral and central neural sensitization may be a contributor to itch intensity and prolonged itch sensations. Peripheral sensitization occurs where a nerve outside of the brain and spinal cord undergoes increased response to pruritogens or algogens more than normal. This may possibly be due to the sensitization of cutaneous mechano- and heat-sensitive C-fibers (Pogatzki-Zahn et al., 2020). It is well demonstrated in pain conditions when peripheral nerves sense pain situations where they normally would not have responded due to reduced threshold of nociceptive neurons. Studies have shown that AD patients have heightened itch and pain intensity which is predominantly self-inflicted (Andersen et al., 2017; Vakharia et al., 2017). Prurigo nodularis (PN), is a skin condition that is characterized by intensely pruritic symmetric nodular lesions and is strongly associated with central diseases of pain. Studies by Stander group demonstrated significant neural sensitization phenomena in skin in PN patients (Ständer et al., 2022). Furthermore our recent findings of high susceptibility of chronic pain neural sensitization disorders in these patients in a large population study suggests the involvement of common neural sensitization factors of itch and pain in these patients (Agelopoulos et al., 2023; Choragudi and Yosipovitch, 2023).

### 2.1. Nerves in peripheral sensitization

When nerve endings in the sensory afferent neurons are stimulated by external stimuli, voltage-gated channels and/or ligand-gated receptors are activated, leading to action potentials. Sensitization of mechano- and heat-sensitive C-fibers, decreased intraepidermal nerve fiber density, loss of C-fiber function, and increased function in Aδ fibers may contribute to peripheral sensitization (Pogatzki-Zahn et al., 2020).

There is increased evidence that some neuropeptides and immune modulators contribute to peripheral sensitization in chronic itch. This type of sensitization leads to an upsurge in neurotransmitter release and neuro-immune-cell interactions, thereby increasing neurogenic inflammation and nociception. Inflammation induced by nerve growth factor (NGF), prostaglandin E2 (PGE2), abnormal innervation induced by inflammation/scratching, and PAR2 activation in the skin, stimulates the upregulation of itch-related receptors and molecules (i.e., substance P, brain-derived neurotropic factor) in the dorsal root ganglia (DRG) (Yosipovitch et al., 2018).

### 2.2. Piezo and interneuron dysregulation

The Piezo protein is expressed in primary sensory neurons and non-neuronal cells and has a role in peripheral nerve sensitization. Elevated piezo 1 in the DRG proximal to peripheral nerve injury results in painful neuropathy (Shin et al., 2023). Free nerve endings and DRG help to transmit itch signals to the dorsum of the spinal cord, where spinal sensitization via interneuron connections transmits itch to the amygdala region of the brain. Piezo 1 channel is known to drive this itch transmission in sensory neurons upon mechanical stimulation (Pereira et al., 2023). In a study on mice, Piezo 1, a mechanically activated ion channel, was found to be selectively expressed by itch-specific sensory neurons. The study further demonstrated that mechanically induced acute and chronic itch is profoundly reduced with a loss of Piezo 1 function in peripheral sensory neurons (Hill et al., 2022).

The transmission of itch via Piezo 1 and 2 channels involves both histaminergic C-afferents and non-histaminergic C and Aδ afferent fibers leading to release of gastrin-release peptide receptor (GRPR) from the dorsal horn of the spinal cord, hence inducing itch alloknesis and elevated itch intensity (Hill et al., 2022; Pereira et al., 2023). Piezo proteins are required for myelin formation because the absence is known to delay myelin development in peripheral neurons (Acheta et al., 2022). Since itch sensitive nerve fibers are unmyelinated or thinly myelinated, damage to myelin may only affect itch sensation in Aδ fibers (Ringkamp et al., 2011). This further confirms that activity in C-type nociceptive fibers may also be involved in non-histaminergic itch.

### 2.3. Transient receptor potential (TRP) channels

TRP Channels are sensory molecules that exhibit diverse functionality and have important roles in itch transmission. They are abundantly expressed in the skin and sensory neurons and are sensitized by a variety of endogenous and environmental stimuli (Sadler and Stucky, 2019). A key example is the sensitization of TRPV1 channels by nerve growth factor (NGF). In this scenario, NGF binds to its receptor TrkA, which ultimately sensitizes TRPV1 on nerves and increases its sensitivity to molecules such as substance P, calcitonin gene-related peptide, and brain-derived neurotrophic factor (Yosipovitch et al., 2018). Several other TRP channels modulate itch through specific activating compounds as well sensitizing agents, contributing to peripheral sensitization (Mahmoud et al., 2022).

### 2.4. Mas-related G-protein coupled receptors

Recently, more evidence has emerged on the role of non-histaminergic mediators in peripheral neural sensitization. The discovery of Mas-related-G-protein-coupled receptors (MRGPRs)subfamily X (MRGPRs) subtypes 1, 2. 3 and 4 and their role as drivers of inflammation, pain, and itch, has increased our understanding of itch signaling and transmission (Guan et al., 2010). Notably, the itch receptors MRGPRX2transmit signals through diverse signaling pathways. Itch is initiated in the periphery when itch-sensing neurons expressing specific membrane receptors detect their corresponding ligands in the dermatome they innervate (Dong and Dong, 2018). Mast cell-specific MRGPRX2 has been shown to be a key modulator of neurosensitization via the nerve-immune cell interaction and mediates non-histaminergic itch (Green et al., 2019; Thapaliya et al., 2021). Pruritogens induce sensory neurons to release substance P, which activates mast cell internalization of MRGPRX2 and degranulation of the human mast cell (McNeil et al., 2015; Shtessel et al., 2021; Raj et al., 2023). The protease receptors (PARS) are also activated by enzymatic cleavage of the extracellular N-terminal of MRGPRX2 leading to enhanced excitability. Interactions between these neuropeptides may contribute to peripheral sensitization in chronic itch. MRGPRA3 in mice is a distinct itch receptor and has been implicated in mechanisms of chronic itch, and research is emerging regarding its associations in neural sensitization. A recent study describes the role of MRGPRA3 in pruritogen-induced allokinesis via mechanosensitive Piezo 2 channels in mice (Liu et al., 2023). Specifically, the investigators demonstrate that Piezo 2 expressed by TRPV1+/MRGPRA3pruritoceptor neurons play a role in pruritogen-induced allokinesis (Liu et al., 2023). The proposed mechanism involves a pruritogen activated PLC-PKCd signaling pathway that sensitizes Piezo 2 channels and ultimately sensitizes MRGPRA3+ pruritoceptors following mechanical stimulation (Liu et al., 2023). MRGPRC11 has similarly garnered attention for its contributions to itch with emerging evidence speaking to its potential role in sensitization. Human MrgprX1 can be activated to induce itch by various substances, including BAM8- 22, and chloroquine. MrgprX1 and MrgprX2 have recently also been implicated in neuropathic and inflammatory pain via mast cell activation and possibly increase neural sensitization to both pain and itch (Green, et al., 2019). MrgprX1 may act as an analgesic by decreasing neuronal activity or as an algesic by increasing neuronal activity in different circumstances and cell types (Tiwari et al., 2016). Recent data from Dong`s group suggests that MRGPRX1 triggers itch sensation by increasing activity of TTX resistant voltage gated sodium channels further contributing to neural sensitization (Tseng et al., 2019). One study points to tick salivary peptides as pruritogens that sensitize TRPV1 through the activation of MRGPRC11/MRGPRX1 (Li et al., 2021). However, further research is needed to better characterize this role.

### 2.5. Neurotrophins

The neurotrophins brain-derived neurotrophic factor (BDNF), nerve growth factor (NGF), and neurotrophin-4 (NT-4) play important roles in itch caused by neuro-immune interactions. Notable effects of these neurotrophins are their role in increased responsiveness of peripheral neurons to normal or sub-threshold afferent stimulations (Misery et al., 2023). BDNF is thought to be released by pruritogen-stimulated skin resident immune cells and binds to trkB which results in sprouting of peripheral nerves (Weihrauch et al., 2023). Similarly, studies have also demonstrated a positive functional relationship between eosinophils expressing BDNF and nerve fiber outgrowth in itch sensitization (Guseva et al., 2020). When BDNF is released, it sensitizes the peripheral neurons to respond beyond the normal threshold to scratching.

NGF regulates nerve development and neuronal sensitivity. It is released in the peripheral nerve endings and binds to trkA receptors. Once bound to trkA receptors on nociceptive C- and Aδ nerve fiber endings, the signal is transported to DRG where gene expression of other neuropeptides and ion channels are increased. NGF has been reported to induce epidermal nerve sprouting leading to increased pain localization and hypersensitivity in chronic itch (Yosipovitch et al., 2007; Buhl et al., 2020). Increase in nerve fiber density has been associated with elevation in NGF and pruritus. The expression of this neurotrophic factor is upregulated in itchy skin conditions such as chronic urticaria, AD and PN and correlates well with itch intensity (Yamaguchi et al., 2009; Buhl et al., 2020; Deng et al., 2022). It is established that NGF sensitizes cowhage, exclusive of histamine-induced itch, suggesting peripheral sensitization of mechano-sensitive Aδ fibers and peripheral itch-sensitive afferents (Yamaguchi et al., 2009). Another neurotrophic factor, NT-4, was reported to strongly correlate with severity of itch in CKD patients and may be an important driver of itch (Sorour et al., 2019; Wala-Zielinska et al., 2022). Overall, there is increasing evidence suggesting the role of neurotrophins in itch peripheral sensitization.

### 2.6. Periostin

The role of the pruritogen periostin in peripheral neural sensitization has been recently reported. Increased periostin levels have been found in chronic itchy skin conditions such as AD, PN, and bullous pemphigoid, and positively correlated with itch severity (Ariëns et al., 2020; Hashimoto et al., 2021; Kamphuis et al., 2022; Sans-de San Nicolàs et al., 2023). Periostin is an intracellular matrix protein thought to be produced by epidermal keratinocytes and dermal fibroblasts, and binds to its receptor integrin α_V_β3, causing immune cells to release itch cytokines IL-4, IL-13, IL-31 and inducing sensory nerve fiber sensitization by direct activation of integrin alpha B receptor (Masuoka et al., 2012; Hashimoto and Yosipovitch, 2019; Izuhara et al., 2019; Mishra et al., 2020).

### 2.7. Phospholipase A2

Phospholipase A2 (PLA2) is one of the most common allergens known and well characterized in venom of hymenoptera insects and some reptiles (Baumann et al., 2018). The evolution of PLA2 and its role in contributing to classical inflammation and itch is attributed to its interaction with autotaxin, thereby producing lysophosphatidic acid (LPA) which is associated with pruritus in cholestatic diseases (Macias et al., 2018). A study by our group has shown a positive correlation between PLA2 genes and itch intensity in AD and psoriasis (Nattkemper et al., 2018). PLA2 may be inducing itch by direct activation of TRPA1 and TRPV1 and transmission of itch signals via the sensory peripheral fibers (Kittaka et al., 2017). The role of PLA2 in maintaining pain and central neural sensitization after neural injury has been demonstrated. Inhibiting spinal PLA2 during painful injury reduced neuronal firing in the spinal cord and elevates intracellular glutamate concentration; thus, suggesting that spinal PLA2 is implicated in spinal mechanisms of neuronal excitability via glutamate signaling, neurotransmitters, or inflammatory cascades (Quindlen-Hotek et al., 2020; Kartha et al., 2021). Although the exact mechanism via which PLA2 cause peripheral and central itch sensitization is not well understood, we propose that its ability to stimulate signal transduction and excitation of itch sensory neurons may contribute to peripheral sensitization. There is need for mechanistic studies to evaluate the specific roles of PLA2 in peripheral and central itch sensitization.

### 2.8. Skin resident immune cells

Activation of immune cells (mast cells, basophils, eosinophils, macrophages, T cells, and keratinocytes) and peripheral sensory fibers release itch mediators which further directly sensitize nociceptors (Nakashima et al., 2019). Sensitization is made possible partly due to the proximity of immune cells and keratinocytes to peripheral sensory c-fibers. Conversely, peripheral nerves are also known to regulate the functions and degranulation of the immune cells to release itch mediators such as cytokines IL-13 and IL-31 (Nakashima et al., 2019). When the sensory C-fibers of the skin are excited by itch mediators released from immune cells, they in turn release itch mediators substance P, NGF, BDNF and NK1, leading to the transmission of neuronal firing to the spinal cord (Nakashima et al., 2019). Taken together, the cycle of itch mediator release from peripheral nerves, immune cells, and keratinocytes, as well as the transmission of itch signals, is responsible for the peripheral sensitization in chronic itch.

## 3. Central neural sensitization in chronic itch

Central neural sensitization in itch refers to the abnormal amplification and dysregulation of itch signals in the central nervous system (CNS), which includes the brain and spinal cord, resulting in heightened responsiveness and sensitivity to itch stimuli. This phenomenon plays an important role in the pathophysiology of chronic itch conditions.

### 3.1. Itch sensitization in the spinal cord

Itch sensation is initially transmitted from the periphery through slow-conducting unmyelinated C-nerve fibers and travels to the spinal cord via dorsal root ganglia, synapsing with second-order projections in the dorsal horn of the spinal cord (Vander Does et al., 2023). When itch signals arrive at the spinal cord, these signals can be modulated by activating or inhibitory interneurons, affecting how itch sensation is perceived (Yosipovitch et al., 2018; Vander Does et al., 2023). Central neural sensitization of itch occurs in the spinal cord either through increased excitatory signaling or through altered inhibitory signaling.

In chronic itch conditions, peripheral inflammation and neuroimmune crosstalk results in the production of cytokines, chemokines, and growth factors, orchestrated in part by Toll-like receptors (TLRs) (Ji, 2015). These peripheral mediators, namely glutamate and brain-derived neurotrophic factor (BDNF), bind to central terminals of primary afferents in the spinal cord and brain stem, leading to increased activity of postsynaptic neurons and increased transmission of itch signals (Ji, 2015). Neurons involved in the itch signaling pathway are excitatory glutamatergic neurons that express vesicular glutamate transporter type 2 (VGLUT2) (Ma, 2014; Dong and Dong, 2018). Nevertheless, genetic knockout studies have demonstrated that glutamatergic transmission is not the only mechanism by which itch signals are propagated, and that neuropeptides such as gastrin releasing peptide (GRP) and natriuretic polypeptide B (NPPB, also known as B-type natriuretic peptide) also play a central role (Dong and Dong, 2018).

Taking a closer look at the pathway, there are two types of itch stimuli, chemical itch stimuli, which are endogenous or exogenous pruritogens, and mechanical itch stimuli, which are typically light tactile stimuli – each functioning through a separate circuit (Sakai and Akiyama, 2020). Chemical itch stimuli activate peripheral itch receptors on primary sensory neurons that relay itch signals through the release of the itch excitatory neurotransmitters glutamate and NPPB. These molecules bind to NPPB receptor A (NPRA) on NPRA-positive spinal neurons, and from there, NPRA-positive or GRP-positive neurons release GRP thereby activating GRP receptor (GRPR)-positive neurons (Yosipovitch et al., 2018; Chen and Sun, 2020). Substance P (SP) binds neurokinin-1 (NK1) receptors in the spinal cord, contributing to the propagation of chemical itch signals in this pathway (Jin and Wang, 2019).

In addition to increased activity and excitability of neurons in the dorsal horn of the spine, neuroinflammation and resultant glial cell activation appears to contribute to central neural sensitization of itch. This phenomenon was first described in chronic pain, by which tissue and nerve inflammation in the periphery activates glial cells (microglia and astrocytes) in the spinal cord and brain via adenosine triphosphate, chemokines, and proteases (Yosipovitch et al., 2020). The activated glial cells induce an intracellular signaling cascade with activation of protein kinases, leading to the release of cytokines (TNF-alpha, IL-1B, and IL-6), chemokines (CCL1 and CXCL1), and growth factors (BDNF) that can directly modulate pain signaling, contributing to neural sensitization. Central sensitization can further propagate the activation of glial cells and amplify central sensitization in a mechanistic loop (Ji, 2015; Li et al., 2021). Reactive astrocytes secondary to chronic itch have been found to play a role in sensitization of itch in the spine and may, in part, explain the amplification and maintenance of itch in chronic itch conditions. Recent mouse model studies have demonstrated that in states of chronic itch, astrocytes in the dorsal horn of the spine become reactive in a STAT-3 dependent manner (Yosipovitch et al., 2018; Shiratori-Hayashi and Tsuda, 2020). The reactive astrocytes then release lipocalin-2, which activates GRPR-positive neurons, enhancing itch sensation via the chemical itch signaling pathway (Koga et al., 2020; Shiratori-Hayashi and Tsuda, 2020; Liu et al., 2023). In a study by our group, overexpression of GRPR-positive neurons in the dorsal horn of the spinal cord (in superficial lamina I and II) was found in primates with idiopathic chronic itch (Nattkemper et al., 2013). The upregulation of GRP and GRPR in the spinal cord can drive hyperactivity of itch signals and central sensitization. Recent studies have identified receptors expressed on astrocytes in the dorsal horn of the spinal cord that may play a role in the activation of astrocytes as well as chronic itch (Shiratori-Hayashi and Tsuda, 2020). In a mouse model study by Liu et al., Toll-like receptor 4 (TLR4) was discovered to be involved in both astrocyte activation and chronic itch (Liu et al., 2016). Other receptors include the IL-33 receptor, ST2, and the chemokine receptor, CXCR3, although further studies exploring the role of these receptors in the context of glial inflammation and chronic itch are needed (Benarroch, 2022). Reactive astrocytes may play a role in central sensitization in the context of mechanical itch, although further studies are needed in this area. In chronic itch patients, where pain is a common symptom, it becomes challenging to determine the extent to which itch or pain contributes to astrogliosis in chronic itch conditions (Shiratori-Hayashi and Tsuda, 2020).

When itch signals reach the spinal cord, they are also modulated by spinal interneurons, with spinal inhibitory interneurons functioning to dull the itch signal relaying pathway. Moreover, dysregulation of spinal inhibitory neurons can lead to hyperactivity of the itch pathway and increased itch (Chen and Sun, 2020). Helix–loop–helix family member B5 (Bhlhb5)-positive spinal inhibitory interneurons can attenuate chemical itch signaling by inhibiting the itch propagating spinal signaling pathways via the release of the opioid peptide neuromodulator, dynorphin, and the inhibitory neurotransmitters GABA and glycine (Yosipovitch et al., 2018; Chen and Sun, 2020). Dynorphin exerts an inhibitory effect on itch in the spinal cord by binding to itch-inhibiting kappa-opioid receptors on neurons that transmit itch sensation. Bhlhb5-positive spinal inhibitory interneurons can also be activated by scratching, pain (relayed by TRPV1 and TRPA1 positive nerves), cooling (relayed by TRPM8 positive nerves), and descending supraspinal inhibitory signals from the periaqueductal gray matter (PAG) (Yosipovitch et al., 2018). Additionally, spinal interneurons expressing galanin, which often overlap with Bhlhb5- and dynorphin-positive neurons, also function in the inhibitory pathway by inhibiting GRPR-positive neurons directly (Chen and Sun, 2020).

Neural sensitization of itch can occur at the level of the spinal cord through the dysregulation of these itch inhibitory circuits. Bhlhb5-positive interneurons co-express dynorphin and somatostatin receptors; peripheral somatostatin can bind to these inhibitory interneurons, and in turn, inhibit the release of dynorphin, GABA, and glycine from Bhlhb5-positive interneurons (Huang et al., 2018; Chen and Sun, 2020). The cumulative effect is “inhibiting the inhibitor” and propagation of uninhibited itch signals. Additionally, neural sensitization of itch can occur by decreasing the descending inhibitory signals from the PAG, with noradrenaline and serotonin playing modulatory roles. The descending pathways from PAG involve the release of norepinephrine that binds to excitatory alpha1-adrenoreceptors on Bhlhb5-positive interneurons and inhibitory alpha2-adrenoreceptors located on central terminals of primary sensory neurons in the dorsal horn of the spinal cord, both of which function to decrease transmission of itch signaling (Yoshimura and Furue, 2006; Gotoh et al., 2011). On the other hand, descending serotonergic signaling mediated by serotonin receptor 5-HT1A, can worsen itch by enhancing GRP/GRPR signaling in the spine (Yosipovitch et al., 2018).

Mechanical itch stimuli such as itch evoked by applying a brush or scratching the skin activate low-threshold mechanoreceptors (LTMR) to propagate itch signaling through a separate circuit than that of chemical itch. After activation of LTMRs in the periphery, itch signals are relayed to spinal urocortin 3 (Ucn3)-positive neurons, some of which also express neuropeptide Y1 receptor (NPY1R) (Pan et al., 2019; Chen and Sun, 2020). NPY1R-positive interneurons play an excitatory role in the mechanical itch pathway and are essential for transmitting mechanical itch signals received from LTMRs (Sakai and Akiyama, 2020). Neuropeptide Y (NPY)-positive inhibitory interneurons have been shown to inhibit mechanical itch signaling in the spinal cord by regulating Ucn3- and NPY1R-positive neurons via NPY signaling and GABA and glycine inhibitory neurotransmitters (Chen and Sun, 2020; Sakai and Akiyama, 2020). Neural sensitization in the spinal mechanical itch pathway is an area of evolving research, although is likely to involve increased excitatory signaling of Ucn3- and NPY1R-positive neurons, or dysfunction of inhibitory NPY-positive inhibitory interneuron signaling. A recent mouse model study found that inhibitory inputs to Ucn3-expressing excitatory neurons are decreased in mice with atopic dermatitis (Pan et al., 2019).

### 3.2. Itch sensitization in the brain

After processing in the spinal cord, itch signals then ascend along the spinothalamic tract and spinoparabrachial pathway up to the thalamus and parabrachial nucleus (PBN), respectively (Vander Does et al., 2023). Itch signals are then transmitted to various areas of the brain associated with itch processing (e.g., the anterior cingulate cortex, the posterior cingulate cortex, the prefrontal area, median raphe nucleus, amygdala, hippocampus, and other brain areas) (Yosipovitch et al., 2018).

Brain imaging studies investigating chronic pain offer valuable insight into the underlying mechanisms and the affected brain regions impacted by prolonged pain sensation, which in turn, can help to elucidate the potential mechanisms of central sensitization in the context of chronic itch. Imaging of central pain sensitization shows evidence of structural gray matter changes in important brain areas associated with pain processing (e.g., thalamus, periaqueductal gray, insula, cingulate, and somatosensory cortices), and neurochemical changes including increased levels of excitatory neurotransmitters (e.g., glutamate) and decreased levels of inhibitory neurotransmitters (e.g., GABA) in key brain regions (Harte et al., 2018). Significant functional brain alterations have also been reported in chronic pain, with increased resting brain network connectivity to pro-nociceptive areas and decreased connectivity to anti-nociceptive areas (Harte et al., 2018). Central neural sensitization is induced and maintained, in part, by glutamatergic signaling to post-synaptic N-methyl-D-aspartate (NMDA) receptors. In the context of pain sensitization, NMDA receptors have been found to play a critical role in increasing nociceptive neuron excitability, with blockage of NMDA receptors preventing and reversing the hyperexcitability of nociceptive neurons (Latremoliere and Woolf, 2009). Furthermore, sustained NMDA receptor-mediated transmission leads to remodeling and influences brain plasticity over the long term (Ji et al., 2018; Bazzari and Bazzari, 2022). Central itch sensitization is likely to follow a similar mechanism.

In chronic itch, central neural sensitization at the level of the brain is associated with increased neural activity and structural and functional changes to itch-associated brain areas (Yosipovitch et al., 2018). Brain imaging studies of chronic itch patients are limited, although current studies show a trend of structural and functional differences in sensory and motor-related brain regions when compared to healthy individuals (Jin and Wang, 2019; Najafi et al., 2020). Furthermore, imaging studies of chronic itch with fMRI demonstrate an overlap in brain regions with chronic pain indicating that central sensitization mechanisms are likely to be similar (Harte et al., 2018; Wang et al., 2018; Zhang L. et al., 2022).

Increased neural activity associated with central sensitization has been reported in the thalamus, insula, amygdala, anterior cingulate cortex (ACC), and somatosensory cortices in chronic itch patients (Yosipovitch et al., 2018). A brain imaging study by Ishiuji et al. (2009) reported hyperactivity in the ACC and insula in AD patients compared to controls. In another brain imaging study, Mochizuki et al. (2015) observed greater activity in regions of the brain associated with motor control and motivation (e.g., supplementary motor area, premotor cortex, primary motor cortex, midcingulate cortex, and caudate nucleus) in chronic itch patients engaged in scratching behavior compared to healthy controls (Mochizuki et al., 2015). Schneider et al. (2008) reported central changes to motor areas in patients with chronic itch, with increased activation of the basal ganglia.

In addition to increased neuronal activity in CNS itch-associated brain regions, brain imaging studies have reported structural changes, particularly, in gray matter density and volume in itch-associated areas. Patients with end-stage renal disease were found to have an increased gray matter density in the brain stem, hippocampus, amygdala, midcingulate cortex, and nucleus accumbens, while significant thinning of gray matter was noted in the insula, ACC, precuneus, and caudate nucleus; these are areas involved in itch processing and inhibition that may explain central sensitization (Papoiu et al., 2014). In a study by Wang et al. (2021) compared to healthy controls, chronic spontaneous urticaria (CSU) patients were found to have a significantly higher gray matter volume in the right premotor cortex, left fusiform cortex, and left cerebellum, with gray matter volume in the left fusiform cortex significantly associated with Urticaria Activity Score 7 (assesses the number of hives and severity of itching in 7 days). Additionally, significant gray matter volume increases were found in the right putamen and right ventral striatum of CSU patients (Wang et al., 2018). These brain regions have been previously linked to itch in functional brain imaging studies, and the observed increase in gray matter volume and density is likely the result of heightened activity in response to chronic itching and scratching.

Brain function changes can also be assessed by fMRI examining the resting state functional connectivity of brain regions, or the areas of the brain that are simultaneously activated or deactivated at rest (Harte et al., 2018). This can provide valuable insights into central sensitization. Alterations in functional connectivity between itch-related regions of the brain have also been reported in imaging studies of chronic itch patients. Compared with healthy controls, the left fusiform cortex in CSU patients demonstrated significantly decreased functional connectivity with the right orbitofrontal cortex, medial prefrontal cortex, premotor cortex, primary motor cortex, and the cerebellum, and increased functional connectivity with the right posterior insular cortex, primary somatosensory cortex, and secondary somatosensory cortex. Other CSU imaging studies have reported decreased resting state functional connectivity between the right ventral striatum and the right occipital cortex, and between the right putamen and left precentral gyrus, as well as changes in resting-state functional connectivity between thalamic regions and other brain areas associated with sensorimotor function and scratching (Wang et al., 2018; Zhang L. et al., 2022).

An imaging study of AD patients found decreased functional connectivity from baseline state to allergen evoked-itch state between itch-related brain regions, namely the insular and cingulate cortices and basal ganglia; the decreased connectivity correlated significantly with increased level of perceived itch. After allergen-induced itch, there was also increased connectivity between the superior parietal lobule and dorsolateral prefrontal cortex (Desbordes et al., 2015). A study of psoriasis patients reported differences in white matter microstructure and functional connectivity compared to healthy controls, particularly in areas that convey itch sensation (Najafi et al., 2020). A study of brachioradial pruritus patients found decreased functional connectivity within the default mode network including the precuneus and the cingulate cortex compared with healthy controls (Dehghan Nayyeri et al., 2022).

Neural activity, structure, and function of itch-associated brain regions in chronic itch patients differ from healthy individuals, potentially indicating central neural sensitization. Further functional brain imaging studies in chronic itch patients are needed to assess changes in areas responsible for itch processing, such as in the amygdala, which shows high functional connectivity in healthy subjects (Mochizuki et al., 2019; Wang et al., 2021).

## 4. Treatments for neural sensitization of itch

There are many neuroimmune-modulating therapies that target itch neural sensitization mechanisms as well as provide relief for patients with chronic itch (Table 1).

**Table 1:** Treatments for neural sensitization and their potential mechanisms.

Therapy	Mechanisms
*GABAergic Drugs*	Bind to voltage-gated calcium channels in the CNS, reducing calcium influx into nerve terminals and the release of itch excitatory neurotransmitters (e.g., glutamate and substance P). Increase GABA inhibitory neurotransmission in the CNS and decrease the release of peripheral substance P and calcitonin gene-related peptide from primary afferent neurons by increasing spinal cord GABA.
*Kappa Opioids*	Correct imbalance in endogenous opioid system to help reduce itch sensitization in the CNS.
*Immunomodulatory Therapies (e.g., Dupilumab, Nemolizumab, JAK Inhibitors)*	Decrease the effect of itch-promoting cytokines IL-4, IL-13, and IL-31 on peripheral itch-conveying sensory nerve fibers, thereby reducing peripheral sensitization. Dupilumab = targets alpha-subunit of the IL-4 receptor, reducing type 2 cytokines IL-4 and IL-13. Nemolizumab = reduces IL-31-induced sensory nerve stimulation. JAK inhibitors (e.g., ruxolitinib, abrocitinib, upadacitinib, baricitinib, tofacitinib, delgocitinib) – target itch-promoting cytokine signaling pathways.
*NMDA Receptor Targets (e.g., Ketamine)*	Inhibit NMDA-dependent excitatory signaling in the periphery (topical application) and centrally (intravenous or inhaled formulations); central-acting formulations may reduce central sensitization by attenuating neural plasticity.
*Antioxidant Flavonoids*	Antioxidant (neutralize free radicals and reactive oxygen species) and anti-inflammatory (decrease pro-inflammatory cytokines and chemokines) effects. Potentially inhibit MRGPRX2-mediated mast cell activation.
*Cognitive Behavioral Therapy*	Decrease stress, maladaptive thoughts, and scratching behaviors leading to reduced itch and affecting brain structure and function.
*Transcranial Magnetic Stimulation*	Modulate neural activity of brain regions via application of a weak magnetic field through the scalp.

### 4.1. GABAergic drugs

Gabapentin and pregabalin are GABAergic drugs that are indicated for neuropathic pain as well as many different chronic itch conditions including uremic pruritus, neuropathic itch, PN, chronic pruritus of unknown origin (CPUO), cutaneous T-cell lymphoma (CTCL), and paraneoplastic itch (Yosipovitch et al., 2018). The mechanism by which these drugs function to reduce itch is likely by decreasing central neural sensitization through the modulation of neurotransmitters (Iannetti et al., 2005). These medications bind to voltage-gated calcium channels in the CNS, reducing calcium influx into nerve terminals and the release of itch excitatory neurotransmitters such as glutamate and substance P (Shavit et al., 2013). Gabapentin and pregabalin also increase GABA inhibitory neurotransmission in the CNS and decrease the release of peripheral substance P and calcitonin gene-related peptide, an important itch mediator, from primary afferent neurons by increasing spinal cord GABA (Fehrenbacher et al., 2003). Activation of PAG GABAergic neurons or inhibition of glutamatergic neurons was found to attenuate scratching behavior in both acute and chronic itch (Samineni et al., 2019).

### 4.2. Kappa opioids

Kappa opioids play an important role in the treatment of chronic itch by targeting imbalances in the endogenous opioid system. As part of the inhibitory circuit in the spinal cord chemical itch pathway, dynorphin is released from Bhlhb5-positive inhibitory interneurons in the spinal cord and binds to κ-opioid receptors (KORs) on GRPR-positive neurons, resulting in the suppression of chemical itch (Yosipovitch et al., 2018). In contrast to the itch-inhibiting KORs, μ-opioid receptors (MORs) are responsible for increased itch. In chronic itch conditions such as uremic pruritus, imbalances in the endogenous opioid system involving itch-activating MORs and itch-inhibiting KORs is linked to neural sensitization (Mochizuki et al., 2019). The KOR agonists nalfurafine and difelikefalin, as well as the mixed KOR agonists/MOR antagonists nalbuphine and butorphanol, function to correct this imbalance and help to reduce itch sensitization in the CNS (Davies and From, 1988; Papoiu et al., 2015; Fishbane et al., 2020a,b; Weisshaar et al., 2022).

### 4.3. Immunomodulatory therapies

Immunomodulatory anti-cytokine therapies may reduce itch neural sensitization through a peripheral effect. IL-4, IL-13, and IL-31 play important roles in the pathophysiology of itch and act on peripheral itch-conveying sensory nerve fibers to induce sensitization (Izuhara et al., 2019; Hashimoto et al., 2020; Sans-de San Nicolàs et al., 2023). Targeting these cytokines has been found to reduce neural sensitization (Yosipovitch et al., 2018). The monoclonal antibody Dupilumab targets the alpha-subunit of the IL-4 receptor, reducing type 2 cytokines IL-4 and IL-13. A recent small-scale study found Dupilumab to not only reduce itch, but also reduce histaminergic and mechanical hyperknesis in AD patients (Hashimoto et al., 2022). The anti-IL-31 receptor alpha monoclonal antibody, Nemolizumab, may reduce IL-31 induced sensory nerve stimulation, thereby reducing peripheral sensitization (Ständer et al., 2022). The Janus kinase (JAK) and signal transducer and activator of transcription (STAT) signaling pathways play an important role in mediating the effects of itch-promoting cytokines IL-4, IL-13, and IL-31 in chronic itch conditions; therefore, use of JAK inhibitors (e.g., ruxolitinib, abrocitinib, upadacitinib, baricitinib, tofacitinib, delgocitinib) potentially offer significant benefits in reducing peripheral itch neural sensitization (Yosipovitch et al., 2018).

### 4.4. NMDA receptor targets

The NMDA-receptor antagonist, ketamine, applied topically has shown efficacy in reducing peripheral nerve hypersensitivity associated with chronic itch (Fowler and Yosipovitch, 2019). Ketamine given intravenously or inhaled differs from the topical formulation in that it has central effects (Fowler and Yosipovitch, 2019). Considering the many biochemical roles that NMDA plays in the CNS, ketamine given in these formulations may reduce central itch neural sensitization by attenuating neural plasticity, although further research is needed in this area.

### 4.5. Antioxidant flavonoids

Flavonoids are compounds that have both antioxidant as well as anti-inflammatory properties – they can neutralize free radicals and reactive oxygen species, decreasing oxidative stress, as well as decrease pro-inflammatory cytokines and chemokines in the periphery (Lee et al., 2020). Furthermore, flavonoids have been reported to inhibit MRGPRX2-mediated mast cell activation in mouse model studies. MRGPRX2 plays an important role in many chronic itch conditions including AD, urticaria, and chronic prurigo (Soares et al., 2023). Collectively, these effects help to decrease peripheral itch sensitization, and potentially central itch sensitization by decreasing oxidative stress and inflammation. There are studies demonstrating the efficacy of flavonoids in reducing itch in chronic itch patients, although further studies are needed to assess the specific mechanisms by which they affect neural sensitization in itch (Zhou et al., 2017; Zhang Y. et al., 2022).

### 4.6. Cognitive behavioral therapy

Non-pharmacologic, stress-reducing psychotherapies such as cognitive behavioral therapy (CBT) can decrease stress, maladaptive thoughts, and scratching behaviors leading to reduced itch (Schut et al., 2016; Mochizuki et al., 2017). Neurobiological studies have demonstrated that psychotherapies such as CBT can affect brain structure and function (Linden, 2006; Felmingham et al., 2007; Desbordes et al., 2012; Hölzel et al., 2013; Huang et al., 2018). Moreover, chronic pain studies have demonstrated structural and functional changes in patients after these interventions, with one study finding reduced brain activity in the right anterior cingulate cortex and parahippocampal gyrus after CBT in patients with irritable bowel syndrome (Lackner et al., 2006; Flor, 2014). In the context of itch, CBT has proven to be an effective add-on therapy for improving itching and scratching in AD patients, improving mental health and overall quality-of-life (Spielman et al., 2015; Schut et al., 2016). Additional studies are needed to assess changes in brain structure and function before and after CBT in chronic itch patients.

### 4.7. Transcranial magnetic stimulation

Transcranial magnetic stimulation (TMS) involves the application of a weak magnetic field through the scalp to affect neural activity. TMS can modulate activity in brain regions involved in the processing of pain, producing an analgesic effect (Mochizuki et al., 2017). However, studies on TMS use in itch are limited. A recent study administered TMS to the contralateral primary and secondary somatosensory cortices and the ipsilateral inferior frontal gyrus after histamine-induced acute itch and found that there was a significant reduction in itch intensity in the primary and secondary somatosensory cortices following TMS therapy (Jones et al., 2019). Given these findings, it would be interesting to further explore the use of TMS in chronic itch and its modulatory role in central neural sensitization of itch.

## 5. Conclusion and future perspectives

Neural sensitization in the context of chronic itch is orchestrated by various signaling pathways and mediators in both the peripheral and central nervous systems. Future research is needed to investigate the crosstalk between itch mediators, immune cells, and sensory nerves as drivers of neural sensitization in chronic itch. Additional studies are also needed to expand on our knowledge of the central mechanisms of itch from the spinal cord to the brain, beginning with understanding the spinal circuits and supraspinal pathways in relation to chemical and mechanical itch pathways. Functional neuroimaging studies assessing brain neural activity, structure, and function are also warranted. As our understanding of the neurobiology of chronic itch deepens, we can expect an expansion in therapeutic options, with the emergence of novel and more effective treatments.

## Author contributions

OM: Writing – original draft. OO: Writing – original draft. RM: Writing – original draft. GY: Writing – review & editing.

## Funding

The author(s) declare that no financial support was received for the research, authorship, and/or publication of this article.

## Conflict of interest

GY serves as an advisory board member for Abbvie, Arcutis, BMS, Cara Therapeutics, GSK, Escient Health, Eli Lilly, Galderma, Kiniksa Pharmaceuticals, LEO Pharma, Novartis, Pfizer, Pierre Fabre, Regeneron Pharmaceuticals, Inc., Sanofi, TreviTherapeutics, and Vifor. GY receives grants/research funding from Eli Lilly, Kiniksa Pharmaceuticals, LEO Pharma, Novartis, Pfizer, Galderma, Escient, Sanofi Regeneron, and Celldex. GY is an investigator for Regeneron Pharmaceuticals, Inc., and Sanofi.

The remaining authors declare that the research was conducted in the absence of any commercial or financial relationships that could be construed as a potential conflict of interest.

## Publisher’s note

All claims expressed in this article are solely those of the authors and do not necessarily represent those of their affiliated organizations, or those of the publisher, the editors and the reviewers. Any product that may be evaluated in this article, or claim that may be made by its manufacturer, is not guaranteed or endorsed by the publisher.
